# Circulating Epstein-Barr virus DNA associated with hepatic impairment and its diagnostic and prognostic role in patients with gastric cancer

**DOI:** 10.3389/fmed.2022.1028033

**Published:** 2022-10-06

**Authors:** Hui-Chan He, Rui Han, Bo-Heng Xu, Chan Huang, Min-Min Li, Cai-Yun He, Wen-Qian Lin

**Affiliations:** ^1^State Key Laboratory of Oncology in South China, Department of Blood Transfusion, Sun Yat-sen University Cancer Center, Collaborative Innovation Center for Cancer Medicine, Guangzhou, China; ^2^Center for Clinical Laboratory, The First Affiliated Hospital of Jinan University, Guangzhou, China; ^3^State Key Laboratory of Oncology in South China, Department of Molecular Diagnostics, Sun Yat-sen University Cancer Center, Collaborative Innovation Center for Cancer Medicine, Guangzhou, China

**Keywords:** Epstein-Barr virus, gastric cancer, liver function, inflammation, diagnosis, prognosis

## Abstract

Epstein–Barr virus (EBV) infection may affect all tissues and organs of the body. Little is known about the impact of this entity on its systematic incorporation in patients with gastric cancer (GC). This study enrolled a total of 113 GC patients with EBV infection (EBVaGC) and 167 GC patients without EBV infection (EBVnGC). It was found that the CRP levels (indicative of inflammatory status) were significantly increased in EBVaGC compared with those in EBVnGC (12.11 mg/L vs. 5.72 mg/L, *P* = 0.008), but WBC and neutrophils counts were similar in both groups (*P* > 0.05). Consistent elevations in the levels of liver enzymes, ALP and GGT, with incompatible alterations in ALT or AST were observed in EBVaGC. Slightly prolonged coagulation indices, PT and INR, and decreased albumin consistently suggested impaired synthesis capability of the liver in EBVaGC (all *P* < 0.05). The level of circulating EBV DNA was positively correlated with the level of GGT, tumor marker CA72-4 and the lymphocyte infiltration in tumor tissues (all *P* < 0.05). Of note, the EBV associated high-lymphocyte infiltrated tissues presented rich CD8 + T cells. Circulating EBV DNA further showed a predictive role in distinguishing EBVaGC from EBVnGC (AUC 0.79, 95% CI 0.73 to 0.85, *P* < 0.001), and was associated closely with better overall survival (HR 0.45, 95% CI 0.21 to 0.96, *P* = 0.039). EBV infection in patients with gastric cancer may be linked to hepatic impairment and immune response. Circulating cell-free EBV DNA is not only a biomarker for the screening of an EBV-related GC subtype but is also an independently prognosis factor for the long-term survival benefit in GC patients.

## Introduction

Epstein-Barr virus(EBV) is a type of herpes virus that may establish a persistent infection in more than 90% of adults and will be asymptomatic (1). EBV-associated gastric cancer (EBVaGC) is a distinct subtype that accounts for 5.1% of gastric cancer (GC) in China (2). Increasing studies have been focused on differential treatment between gastric cancer, with or without EBV infection (3, 4). *In situ* hybridization of the EBV-encoded small RNA (EBER) transcripts have been known as the gold standard method for detecting EBV-associated carcinomas (5). Whether the circulating cell-free EBV DNA could be used as a biomarker for the detection and/or prediction of prognosis in patients with EBVaGC warrants further investigation. For this reason, the quantitative testing of EBV DNA and the identification of viral genome in tissue and in plasma has become essential in the current study (6).

Current evidence indicates a systemic effect of EBV infection on the liver, hematologic, and immune systems in EBV-related diseases but this is rare in malignancies (7–9). For example, some of these patients who displayed symptoms of EBV infection were reported to have more lymphocytes than normal, fewer than normal neutrophils, and abnormal liver enzymes (10). It has been documented that hepatitis is common in EBV-infected patients but is usually transient and self-limiting (11); cholestatic liver disease and chronic hepatitis due to EBV have been described in some previous case-report studies but are considered rare complications (12). Severe hepatitis or acute liver failure caused by EBV infection have also been observed (13, 14). The presentation of EBV infection in the liver may range from mild and transient elevation of aminotransferases to acute hepatitis, and can even lead to acute liver failure. Nevertheless, in most cases, there is no EBV DNA in non-neoplastic and normal liver tissue, indicating that there is a systemic effect caused by EBV infection (15).

EBV infection may affect all tissues and organs of the body (16, 17). However, little is known about the impact of this entity on its systematic incorporation in patients with EBVaGC because of its rarity. Whether and how EBV infection affects the biological systems in patients with GC has not yet been reported. An understanding of the systemic effect of EBV may facilitate optimal decision-making in anti-tumor treatment. In this study, the differences in the clinical indices of multiple important systems between patients with EBVaGC and EBV negative-gastric cancer (EBVnGC) were compared. The aim was to investigate the influence of this entity in patients with GC, with the intention to improve the treatment of patients with EBVaGC.

## Materials and methods

### Research subjects

**FIGURE 1:**
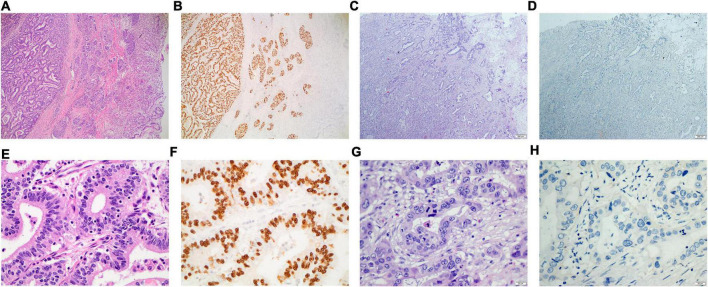
Eosin hematoxylin staining and In situ hybridization of EBERs for the primary tumor tissue of gastric cancer with or without EBV infection. **(A,B)** for the gastric cancer with EBV infection (×40); **(C,D)** for the gastric cancer without EBV infection (×40); **(E,F)** for the gastric cancer with EBV infection (×400); **(G,H)** for the gastric cancer without EBV infection (×400).

The clinical data of 167 gastric cancer patients without EBV infection and 113 gastric cancer patients with EBV infection admitted to Sun Yat-sen University Cancer Center (SYSUCC) from January 2016 to July 2022 were retrospectively analyzed. The pathological diagnosis and laboratory results of routine blood analyses, biochemical tests, blood coagulation tests, and polymerase chain reaction (PCR)-based tests of the EBV DNA level were included. Included patients were histopathologically diagnosed with gastric cancer by two independent pathologists. The EBV status of all the enrolled patients was confirmed using EBER staining in the primary tumor tissue, where patients that were EBER-positive and negative were referred to as EBVaGC and EBVnGC (Figure 1), respectively. All patients provided written informed consent for their information and for the storage of tumor tissues and blood samples for scientific research in the hospital database of Sun Yat-sen University Cancer Center (SYSUCC). The Institutional Review Board of SYSUCC approved this study (SL-B2022-440-01).

### Experimental method for Epstein–Barr virus-encoded small RNA staining in tissue

The detection of the EBER in tissue was conducted in the Department of Pathological department at SYSUCC according to the manufacturer’s protocol with the CD8 marker (ZSGB-BIO, ISH-7001). In brief, add 100 μL of blocking solution and incubate for 10 min at room temperature in the dark, and then dehydrate in 75%, 95% and 100% gradient ethanol for 2 min each; add 100 μL of gastric enzyme working solution and incubate at 37°C for 10 min and then dehydrate in 75%, 95% and 100% gradient ethanol for 2 min each; add 8 μl digoxigenin labeled EBER probe, cover the slide, hybridize and incubate overnight at 37°C in the *in situ* hybridizer (Leica, ST500-24), and then immerse in PBS buffer for 10 min to remove the coverslip and rinse with PBS buffer three times for 2 min each; add 30 μl HRP labeled anti digoxin antibody and incubate at 37°C for 30 min, and then rinse with PBS buffer three times for 2 min each; add an appropriate amount of freshly prepared DAB solution and incubate at room temperature for 10 min, and then rinse with water and counterstain with hematoxylin staining solution for 10 s. Finally, we observed the staining in the nucleus under the microscope after sealing with coverslip.

### Experimental method for Epstein–Barr virus DNA detection in tissue and plasma

The detection of the EBV DNA load in plasma and in tissue was conducted in the Department of Molecular Diagnosis at SYSUCC using an EBV DNA quantitative detection kit (No. 3400973, Hunan Shengxiang Biotechnology Co., Ltd., China). The details of the detection of the EBV DNA load in the plasma and tumor tissue are available in our previous study (2). For the detection in plasma, 3 mL of peripheral blood was collected from the patients in the presence of the anticoagulant, ethylene diamine tetra acetic acid (EDTA), which was then centrifuged at 3,000 rpm for 5 min. The EBV genome was detected using real-time quantitative PCR, which measured the absolute quantitative value using the standard curve defined by four gradients of 10^3^, 10^4^, 10^5^, and 10^6^ copies/mL of EBV DNA. Samples were analyzed using a Roche Light Cycler 480 real-time fluorescent PCR machine, which was operated strictly in accordance with the reagent and instrument specifications.

The detection of EBV DNA in primary tumor tissue was performed using the abovementioned PCR procedure for plasma. The tumor cell content was assessed using hematoxylin–eosin (HE) staining before DNA isolation. DNA from formalin-fixed and paraffin-embedded (FFPE) sections was extracted using the QIAamp DNA FFPE Tissue Kit (56404, Qiagen), according to the manufacturer’s instructions. The input DNA was 100 ng for each detection.

### Assessment of lymphocyte infiltration and CD8 staining

The density of the lymphocyte infiltration in tumors was assessed by pathologist. A condition that is characterized by prominent lymphocytic infiltration was defined as high-lymphocyte infiltration; otherwise it was defined as low-lymphocyte infiltration (2, 18). The staining was performed according to the manufacturer’s protocol with the CD8 marker (ZSGB-BIO, ZA-0508). In brief, the sections were incubated with primary antibodies to CD8 (1:100) overnight at 4°C and then analyzed with streptavidin peroxidase detection system (Maixin Technology Co., Ltd., China).

### Statistics

The quantitative data were analyzed using the *t* test or Mann–Whitney *U* test. A chi-squared test was used for comparing categorical data between groups, and the Pearson’s correlation coefficient was used to measure the strength of the linear relationship between two numerical variables. Spearman’s Rank correlation coefficient is used to summarize the direction of a relationship between the rankings of two variables. Receiver Operating Characteristic (ROC) curve analysis was used to evaluate the diagnostic value of each index. For univariate analysis, the Kaplan–Meier method is used to estimate the survival curve for predicting the overall survival (OS), and the differences between two groups were performed by the Log rank test. Multivariate survival analyses were conducted with the Cox’s proportional hazards model. A two-sided *P* value of 0.05 or lower was considered statistically significant. Data were analyzed using the SPSS 17.0 software (SPSS, Chicago, IL, United States) and R version 4.0.3.

## Results

### Comparison of circulating inflammatory markers between Epstein–Barr virus-infected and non-infected patients with gastric cancer at admission

It is important to study the associations of circulating inflammatory markers and EBV infection in patients with GC at admission before treatment. We therefore focused on several indices including C-reactive protein (CRP), platelet (PLT), white cell count (WBC), and several types of inflammatory cells in peripheral blood (Table 1). Sharply increased levels of CRP were observed in EBVaGC patients. The third quartile of CRP in EBVaGC was 14.28 mg/L, whereas it was 4.57 mg/L in EBVnGC, which obviously exceeded the normal range of 5 mg/L. We also found significant differences in the platelet (PLT) counts between these two groups. The PLT in 51.4% EBVaGC exceeded the reference level of 300 × 10^9^/L, whereas only 21.6% EBVnGC exhibited abnormal PLT counts. Unlike the parameters mentioned above, the WBC, neutrophil, lymphocyte, and monocyte counts did not increase in EBVaGC (*P* > 0.05).

**TABLE 1:** Comparison of laboratory testing between the patients with and without Epstein–Barr virus and gastric cancer.

Parameters^a^		Gastric cancer			*P*-value^b^
			
	EBER(-)		EBER(+)		
			
**Inflammatory status**					
CRP	166	1.59 (0.63, 4.57)	99	2.52 (0.88, 14.28)	**1.17 × 10^–3^**
PLT	167	234 (186, 288)	109	301 (227, 401)	**9.08 × 10^–7^**
WBC	167	6.37 (5.19, 8.51)	108	6.38 (5.02, 8.11)	0.960
NE%	167	0.60 (0.53, 0.70)	109	0.63 (0.54, 0.71)	0.615
LY%	167	0.28 (0.20, 0.35)	109	0.26 (0.19, 0.34)	0.464
MO%	167	0.07 (0.06, 0.09)	109	0.07 (0.06, 0.08)	0.411
**Liver function**					
ALT	167	14.40 (10.70, 20.90)	99	16.4 (9.6, 22.7)	0.391
AS/AL	167	1.11 (0.91, 1.40)	99	1.21 (0.90, 1.56)	0.415
AST	167	16.70 (14.60, 20.50)	99	18.00 (14.50, 22.40)	0.143
ALP	167	69.90 (56.70, 84.00)	99	75.00 (62.80, 94.20)	**0.025**
GGT	167	18.90 (13.80, 27.00)	99	22.80 (16.00, 35.70)	**0.012**
LDH	167	148.3 (129.10, 170.70)	99	160.70 (132.20, 190.20)	**0.032**
ALB	167	41.70 (39.10, 43.60)	99	40.70 (36.70, 42.90)	**0.013**
GLOB	167	27.13 (24.50, 29.60)	99	28.63 (25.80, 31.79)	**2.87 × 10^–3^**
A/G	165	1.53 (1.39, 1.69)	96	1.43 (1.22, 1.63)	**3.94 × 10^–4^**
TBA	167	2.70 (1.40, 4.60)	99	3.80 (2.34, 7.50)	**2.42 × 10^–4^**
TBIL	167	9.80 (7.60, 12.90)	99	8.60 (6.10, 11.10)	**5.92 × 10^–3^**
**Coagulation function**					
PT	166	10.80 (10.40, 11.43)	107	11.4 (11.10, 11.80)	**1.61 × 10^–7^**
PT%	166	106.50 (94, 120.40)	107	99.90 (92.00, 112.40)	**0.011**
INR	166	0.94 (0.91, 0.99)	107	0.99 (0.96, 1.03)	**1.65 × 10^–7^**
APTT	166	25.95 (23.5, 28)	107	25.10 (23.90, 27.80)	0.676
TT	166	18.50 (17.68, 19.33)	107	17.50 (16.70, 18.40)	**5.52 × 10^–9^**
Fbg	166	2.89 (2.42, 3.36)	107	3.50 (2.82, 4.33)	**7.41 × 10^–8^**
**Tumor biomarker**					
CA19-9	166	11.27 (5.65, 21.13)	106	8.91 (3.65, 15.31)	**0.050**
CA72-4	165	1.74 (1.12, 3.94)	102	2.24 (1.01, 4.05)	0.893
CEA	166	2.27 (1.37, 4.09)	106	2.62 (1.59, 4.69)	0.294

^a^Quantitative data of laboratory testing are expressed by median and interquartile range, and the Mann–Whitney *U* test was used for pairwise comparisons between groups. ^b^Bold values denote statistical significance at the *P* < 0.05 level.

### Alterations in biochemical markers in the hepatic system associated with Epstein–Barr virus infection in gastric cancer

We subsequently evaluated the association of biomarkers with respect to hepatocyte integrity, cholestasis, and liver synthetic function with EBV infection in patients with GC (Table 1). The levels of alkaline phosphatase (ALP), gamma-glutamyl transferase (GGT), and lactate dehydrogenase (LDH) were consistently elevated in EBVaGC. However, the AST and ALT levels did not show obvious elevation in EBVaGC (*P* > 0.05). Decreased albumin (ALB) and the albumin to globulin (A/G) ratio were observed with increased globulin (GLOB). In addition, increased total bile acids (TBA) and decreased total bilirubin (TBIL) were found in EBVaGC.

### Change in coagulation indices in Epstein–Barr virus-infected and non-infected patients with gastric cancer

We also considered whether the EBV infection in patients with GC affected the coagulation function (Table 1). The coagulation index, prothrombin time (PT), and international normalized ratio (INR) were slightly prolonged in EBVaGC compared with that in EBVnGC. Fibrinogen (Fbg) was slight elevated in EBVaGC, which was comparable to the increased CRP and PLT in EBVaGC. The thrombin time (TT) was slightly shorter, while the activated partial thromboplastin time (APTT) did not show statistical alteration. All these values were within reference range in almost all of the EBVaGC and EBVnGC patients.

### Correlations among the hub EBVaGC-associated laboratory biomarkers

**FIGURE 2:**
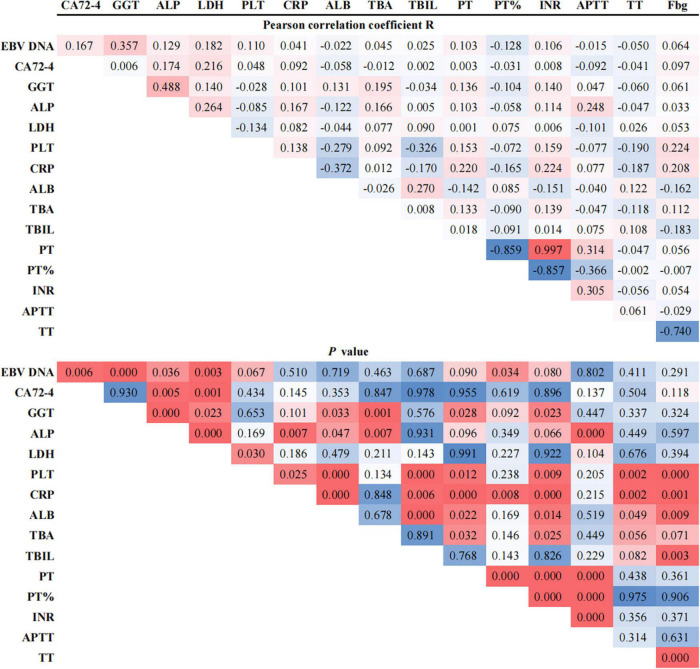
Pearson correlation coefficients between EBV DNA and potentially associated laboratory parameters.

Correlations were determined among the biological biomarkers associated in EBV infection in patients with GC. The circulating cell-free EBV DNA level was positively correlated with the levels of tumor biomarker, CA72-4, and liver enzymes, GGT and LDH, and negatively correlated with the coagulation index, PT% (*P* < 0.05, Figure 2).

### High levels of Epstein–Barr virus DNA in plasma and biopsy tissue of EBVaGC patients

Using PCR analysis, the circulating cell-free EBV DNA was detectable in 71 (62.8%) of the 113 EBVaGC patients as well as in 17 (10.0%) of the 167 EBVnGC patients. The detectable rate and the level of plasma EBV DNA were higher in EBVaGC than in EBVnGC patients (*P* = 0.026) (Table 2). The peak of plasma EBV DNA in EBVaGC reached 3.99 × 10^6^ copies/mL, while none of the plasma EBV DNA in EBVnGC exceeded 250 copies/mL. We further evaluated whether there was viral infection in the primary tumor of the 17 EBVnGC with detectable plasma EBV DNA, and thereby tested the viral DNA in primary tumors of 35 EBVnGC patients (including the abovementioned 17 cases, and 18 cases without detectable EBV DNA in the plasma). None of the 35 EBVnGC had detectable EBV DNA in primary tumors, suggesting that the EBV genome in plasma may not originate from the tumor cells.

**TABLE 2:** Association between tumor EBV-encoded small RNA (EBER) and plasma Epstein–Barr virus (EBV) DNA.

Plasma EBV DNA	Tumor EBER		*P*-value
		
	Positive (*N* = 113)	Negative (*N* = 167)	
Mean value^a^	99,400 ± 44,181	11.98 ± 40.07	**0.026**
Detectable	71 (62.8%)	17 (10.2%)	**1.27 × 10^–20^**
Not detectable	42 (37.2%)	150 (89.8%)	

^a^Absolute quantification of EBV DNA in plasma is expressed by mean ± standard deviation, and the *t* test was used for pairwise comparisons between groups. ^b^Bold values denote statistical significance at the *P* < 0.05 level.

We also tested the EBV DNA in tumor tissues from 35 EBVaGC patients. As expected, all the EBVaGC had detectable EBV DNA in the primary lesions and metastatic lymph nodes, with a very high mean level in the primary lesion (1.90 × 10^5^ copies) (Table 3). In addition, the EBV DNA load in tumor tissue of EBVaGC was positively correlated with tumor content (Pearson correlation coefficient *R* = 0.398, *P* = 0.001). When compared with the primary lesion, the EBV DNA load was not elevated in metastatic lymph nodes, and the tumor content in metastatic lymph nodes was substantially lower (Table 4). However, the correlation between EBV DNA in plasma and *in situ* in primary tumor tissue was not significant (Pearson correlation coefficient *R* = 0.063, *P* = 0.719).

**TABLE 3:** Epstein–Barr virus (EBV) DNA in the primary and metastatic lymph nodes of patients with gastric cancer with EBV infection.

EBVaGC cases	EBV DNA copy number^a^		
	
	Primary lesion	Metastatic lymph node	Metastatic lymph node
1	1,260,000	/	/
2	1,040	/	/
3	30,400	24,000	1,650
4	134,000	21,700	20,000
5	88,000	/	/
6	159,000	3,150	/
7	172,000	81,800	/
8	150,000	69,200	/
9	104,000	/	/
10	45,000	305,000	360,000
11	1,300,000	85,100	73,600
12	167,000	/	/
13	1,140	218,000	/
14	411,000	50,100	93,700
15	18,000	/	/
16	20,200	/	/
17	27,100	506	/
18	1,650	/	/
19	85,900	/	/
20	105,000	24,700	13,200
21	28,400	6,940	20,600
22	260,000	207,000	288,000
23	142,000	/	/
24	298,000	151	46,100
25	65,200	/	/
26	14,800	5,620	71
27	202,000	322	/
28	46,300	28,000	/
29	444,000	36,200	10
30	271,000	/	/
31	42,600	/	/
32	411,000	/	/
33	27,300	/	/
34	95,000	/	/
35	30,700	/	/

^a^The EBV DNA copy number quantification by real-time polymerase chain reaction is shown in absolute quantification with the unit of copies. The EBV DNA copy number in tissue is found to correlate with the tumor content in this study, and thereby the range of EBV DNA copies in metastatic lymph node will be very wide.

**TABLE 4:** Comparison of the Epstein–Barr virus (EBV) DNA between primary lesions and metastatic lymph nodes.

Parameters^a^	Primary lesion (*N* = 35)	Metastatic lymph node (*N* = 29)	*P*-value^b^
EBV DNA copy	190,000 ± 298,000	71,900 ± 10,100	**0.033**
Tumor content (%)	72% ± 24%	40% ± 28%	**1.42 × 10^–5^**

^a^Absolute quantification of EBV DNA in plasma and tumor content are expressed by mean ± standard deviation, and the t test was used for pairwise comparisons between groups. ^b^Bold values denote statistical significance at the *P* < 0.05 level.

### Lymphocyte infiltration in Epstein–Barr virus-infected and non-infected patients with gastric cancer

**FIGURE 3:**
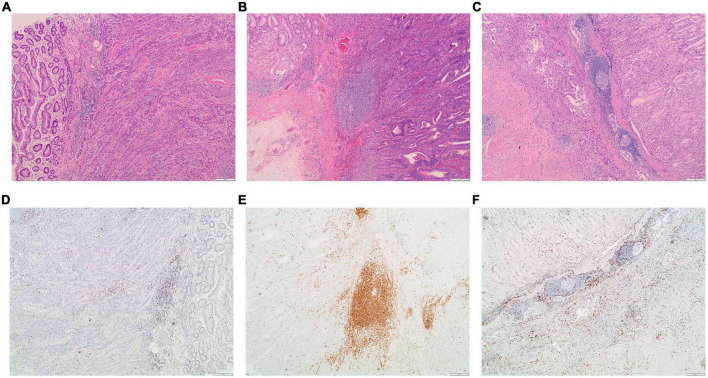
Examples of the morphology and CD8 staining for lymphocyte infiltration in EBVaGC patients. **(A)** Representative images for low-lymphocyte infiltrated tumor; **(B,C)** representative images for high-lymphocyte infiltrated tumors. The corresponding images for the staining of CD8 in the low-lymphocyte infiltrated tumor **(D)** and in high-lymphocyte infiltrated tumors **(E,F)**.

High-lymphocyte infiltration was present in 63.5% EBVaGC cases as compared to 36.5% EBVnGC (*P* = 0.001, Table 5). Of note, the high-lymphocyte infiltration samples presented rich CD8 + T cells. Representative images depicting the high- and low-lymphocyte infiltration in tumors were shown in Figure 3. The lymphocyte infiltration showed a positive correlation with the EBV DNA load in tumor as well as in plasma. Significantly higher EBV DNA load in tumor tissue and in plasma were detected in the high-lymphocyte infiltration tissue (*P* = 0.011 and 0.002). There is a significant positive association between lymphocyte infiltration and EBV DNA load in primary tumor as well as in plasma (with the corresponding Spearman rank correlation coefficient of 0.355 (*P* = 0.009) and 0.302 (*P* = 8.84 × 10^–4^).

**TABLE 5:** Association between Epstein–Barr virus (EBV) status and lymphoid infiltration in primary tumor of gastric cancer.

Parameters	Lymphocyte infiltration		*P*-value^b^
	
	Low	High	
**EBERs staining in tissue**			
Negative	47 (66.2%)	19 (36.5%)	**0.001**
Positive	24 (33.8%)	33 (63.5%)	
EBV DNA in tumor^a^	49,200 (30,400, 172,000)	202,000 (100,225, 395,250)	**0.011**
EBV DNA in plasma^a^	0.0 (0.0, 0.0)	0.5 (0.0, 531.25)	**0.002**

^a^Quantitative data of laboratory testing are expressed by median and interquartile range, and the Mann–Whitney *U* test was used for pairwise comparisons between groups. ^b^Bold values denote statistical significance at the *P* < 0.05 level.

### Diagnostic performance of circulating cell-free Epstein–Barr virus DNA in EBV-associated gastric cancer

**FIGURE 4:**
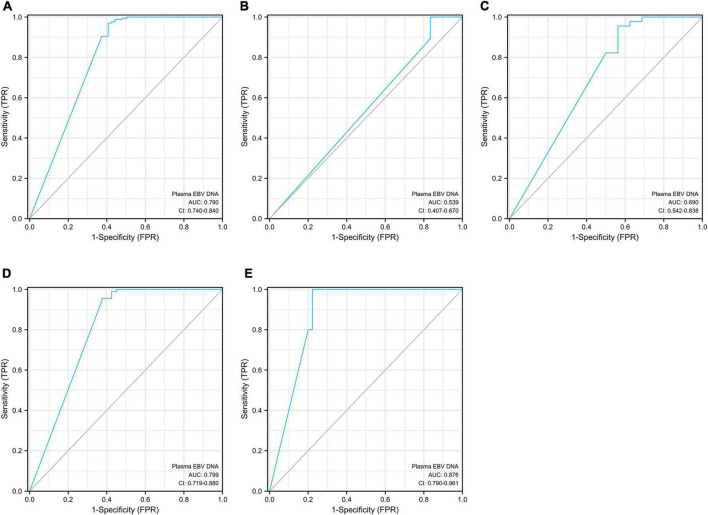
Area under the curve (AUC) for circulating cell-free EBV DNA in distinguishing EBVaGC from EBVnGC. Panel **(A)** for the total gastric cancer; panel **(B)** for the stage TNM I subpopulation; panel **(C)** for the stage TNM II subpopulation; panel **(D)** for the stage TNM III subpopulation; panel **(E)** for the stage TNM IV subpopulation.

The plasma EBV DNA load yielded a total area under the curve (AUC) of 0.79 in distinguishing EBVaGC from EBVnGC (*P* < 0.001, Table 6 and Figure 4A). The cutoff with the best Youden index was 143 copies/mL, which had a sensitivity and specificity of 59.3% and 97.0%. When considering the potential influence of Lauren classification and TNM (Tumor Node Metastasis) stage, we divided the gastric cancer cases into different subgroups (Table 6). In the different Lauren classifications, the performance of plasma EBV DNA showed the best performance in mixed-type GC, with an AUC of 0.84 (*P* ≤ 0.001). Of the different TNM stages (Figures 4B–E), plasma EBV DNA at stage IV performed better than other TNM-stage subgroups, which had an AUC of 0.88 (*P* = 0.006).

**TABLE 6:** Diagnostic performance of circulating cell-free Epstein–Barr virus (EBV) DNA for EBV-associated gastric cancer.

Cutoff value of plasma EBV DNA	Area under the curve (95% CI)	*P*-value^a^
Total gastric cancer	0.79 (0.73–0.85)	**1.70 × 10^–6^**
**Lauren classification**		
Intestinal type	0.73 (0.62–0.85)	**1.43 × 10^–4^**
Diffuse type	0.79 (0.68–0.90)	**3.18 × 10^–6^**
Mixed type	0.84 (0.75–0.93)	**7.96 × 10^–8^**
**TNM staging**		
I	0.54 (0.33–0.74)	0.104
II	0.69 (0.52–0.86)	0.087
III	0.80 (0.70–0.90)	**0.049**
IV	0.88 (0.77–0.98)	**0.006**

^a^Bold values denote statistical significance at the *P* < 0.05 level.

### Prognostic value of circulating cell-free Epstein–Barr virus DNA for overall survival

**FIGURE 5:**
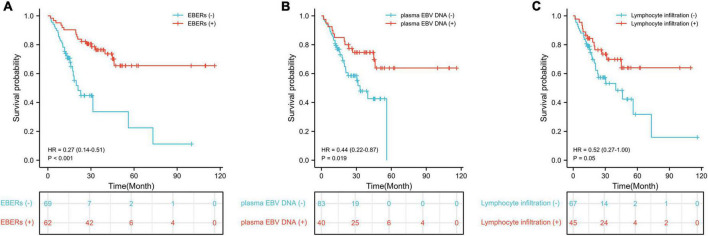
Kaplan–Meier survival estimates of overall survival. **(A)** Survival curve according to EBERs staining; **(B)** survival curve according to plasma EBV DNA; **(C)** survival curve according to lymphocyte infiltration.

In the univariate analysis, differentiation, TNM stage and the status of EBERs staining, EBV DNA in plasma and tumor lymphocyte infiltration were associated with the OS, and the EBV DNA-positive in plasma was related independently to the OS in multivariate analyses (Table 7). The overall survival improved to a statistically significant degree in the group with positive EBERs staining, detectable plasma EBV DNA and high-lymphocyte infiltrated cases in the Kaplan–Meier estimates (Figure 5).

**TABLE 7:** Univariate and multivariate analyses for the overall survival in gastric cancer patients.

Parameters	Hazard Ratio (95%CI)	*P*-value^a^
**Univariate analysis**		
Age	0.99 (0.96–1.02)	0.384
**TNM staging**		
I-II	Ref	
III-IV	4.08 (1.25–13.33)	**0.020**
**Differentiation**		
Poorly	Ref	
Moderately and well	0.39 (0.17–0.91)	**0.029**
**Gender**		
Male	Ref	
Female	0.34 (0.17–0.67)	**0.002**
**EBERs staining *in situ***		
Negative	Ref	
Positive	0.18 (0.09–0.39)	**< 0.001**
**EBV DNA in plasma**		
Not detectable	Ref	
Detectable	0.42 (0.20–0.89)	**0.023**
**Lymphocyte infiltration**		
Low	Ref	
High	0.54 (0.27–1.07)	0.079
**Multivariate analysis**		
**EBV DNA in plasma**		
Not detectable	Ref	
Detectable	0.45 (0.21–0.96)	**0.039**
**TNM staging**		
I-II	Ref	
III-IV	3.79 (1.16–12.41)	**0.028**

^a^Bold values denote statistical significance at the *P* < 0.05 level.

After adjusted by confounding factors, the patients with detectable plasma EBV DNA was associated closely with better overall survival in multivariate analysis (HR 0.45, 95% CI 0.21 to 0.96, *P* = 0.039; Table 7).

## Discussion

This is the first attempt to correlate EBV infection in the patients with gastric cancer and the anomalies in laboratory testing. This study revealed that EBVaGC is associated with multiple alterations in the circulating inflammatory response, coagulation and liver function, specifically with elevated levels of CRP, PLT, GGT, and ALP and decreased of albumin and A/G ratio. This study further demonstrated that EBV infection was closely linked with high density of the tumor lymphocyte infiltration in GC patients. Of note, quantitative real-time DNA amplification of cell-free EBV genome is an alternative option for screening GC cases that are EBV-related and predictive biomarker for the prognosis of GC cases.

Alteration in liver function is one of the most common problems encountered in clinical practice. Failure to address and monitor abnormal liver enzymes can lead to deleterious results (19). Primary EBV infection usually occurs in young children and adolescents and is frequently asymptomatic (20). This infection has been associated with mild hepatitis, which may cause moderate and transitory increase of liver enzymes (21, 22). Alterations in liver enzyme levels encountered in clinical practice can be divided into hepatocellular-predominant and cholestatic-predominant alterations. Individuals with primary EBV infection may be commensurate with substantially elevated levels of ALT and AST, indicating a hepatocellular dominant injury (7). EBV-driven acute liver injury can also be associated with an acute cholestatic hepatitis, but not frequently reported (23, 24). Notably, we found that the liver dysfunction in EBVaGC was characterized by increased levels of ALP and GGT. Cholestatic injury is defined as the disproportionate elevation of ALP, compared with AST and ALT levels (25). ALP is widely distributed but not specific. GGT is merely present in hepatocytes and biliary epithelial cells. Measurement of serum GGT provides a very sensitive indicator for the presence or absence of hepatobiliary injury (26). GGT is helpful in differentiating physiologic elevation of ALP from hepatobiliary disease (27). In the current study, an elevated ALP level of hepatic origin can be confirmed by elevation of GGT (25). In the EBVaGC patients, an elevated ALP level along with an elevated GGT level may suggest cholestatic-predominant liver injury. A positive correlation was also observed between the levels of GGT and circulating cell-free EBV DNA load in EBVaGC patients in the current study. Since the most common causes of liver injury have been excluded in patients with gastric cancer, viral infection should be considered in the etiology of alterations in ALP and GGT in EBVaGC.

Although the levels of ALP and GGT are commonly referred to as liver function tests, they usually reflect hepatocyte integrity or cholestasis rather than liver function (28). In addition to ALP and GGT, PT or INR are valuable measures of the liver’s ability to synthesize fibrinogen and vitamin K–dependent clotting factors: factors II (prothrombin), VII, IX, and X. The current study findings showed slightly prolonged PT and INR and decreased albumin in EBVaGC patients, consistently indicating mild impairment of the synthetic capability of the liver (29). A change in albumin level or PT may be associated with a decrease in liver functioning mass, although neither is specific for liver disease (28). Thus, our findings suggest that elevated GGT and ALP, decreased albumin and A/G ratio, and prolonged PT and INR could work as liver biomarkers to indicate the presence of liver impairment in the context of EBVaGC. However, additional studies are needed to accurately characterize this possible correlation.

Although a tendency toward decreased platelets is frequently observed in acute EBV infection (30), we noted a substantially increased level of PLT in EBVaGC. In a recent study on nasopharyngeal carcinoma and EBVaGC, EBV was reported to induce F3-mediated PLT aggregation that inhibited the antitumor function of natural killer cells (31). In the current study, we also noted a tendency for an elevation in inflammatory CRP in EBVaGC. Nevertheless, the increased CRP was incompatible with increased WBC, neutrophil, or monocyte counts. A high CRP level in the blood can be a sensitive marker of inflammation. Host inflammatory response to virus infection may also result in the release of PLT-activating mediators and a pro-oxidative and pro-coagulant environment, which favors PLT activation or increased D-dimers (32, 33). The exact role of elevated PLT and CRP in EBVaGC is still unclear.

Approximately 5% of Asian gastric cancer patients harbor EBV (2). There are batteries of screening tests for this specific type of GC since it is scarce and insensitive to EBER staining, and partial EBV genome loss (34). Molecular testing is increasingly important in the diagnosis and monitoring of GC patients affected by EBV. The current study showed that, using PCR, the detection of EBV DNA in the primary tumor could be an alternative option for the diagnosis of EBVaGC. The detection of cell-free EBV DNA in the plasma presents a new possibility for the screening of the specific EBVaGC type and an independently prognostic factor for the total GC cases. EBV PCR is a non-invasive laboratory test that helps in identifying EBV infection. Reliable procedures for the whole diagnosis of EBVaGC in practice require not only detection of the gene products in biopsy tissues, but also the monitoring of the viral genome at the level of the peripheral blood. *In situ* hybridization has long been considered the gold standard for detecting tumor-associated viral infection, and EBV viral load assays are now being adopted for clinical evaluation in affected patients (35), especially in cases where the clinical presentation is atypical. Significant survival advantages observed in the patients that are EBV-infected and high-lymphocyte infiltrated suggested potential of benefit from immunotherapy, which warrants further clinical investigation. Prospective trials with outcome data are urgently needed to better understand the prognostic value of cell-free EBV DNA in gastric cancer.

This study has some limitations to be acknowledged. The current study is not a multicenter prospective cohort study. Additionally, the study sample size was relatively small. Moreover, we could not analyze the relationship between EBV DNA load and the expression levels of CD8 due to the unavailability of sufficient GC tissues. Large-scale multicenter studies are needed in future to further validate these results.

In conclusion, our data provide a new view to the influence of EBV infection in liver impairment and immune response in the context of GC, which can aid in directing the subsequent therapeutic work-up. Circulating EBV DNA is a feasible biomarker for screening EBV-infected subtype of gastric cancer as well as for predicting the patients’ prognosis in GC cases.

## Data availability statement

The original contributions presented in this study are included in the article/supplementary material, further inquiries can be directed to the corresponding author/s.

## Ethics statement

The studies involving human participants were reviewed and approved by the Institutional Review Board of Sun Yat-sen University Cancer Center. The patients/participants provided their written informed consent to participate in this study.

## Author contributions

M-ML, W-QL, and C-YH: conception and design. H-CH, C-YH, RH, and B-HX: provision of study material or patients. H-CH, B-HX, RH, and C-YH: collection and assembly of data. H-CH and C-YH: data analysis and interpretation. All authors wrote the manuscript and approved the final version of the manuscript.

## Abbreviations

A/G ratio: albumin globulin ratio
ALB: albumin
ALP: alkaline phosphatase
ALT: alanine aminotransferase
APTT: activated partial thromboplastin time
AST: aspartate aminotransferase
CRP: C-reactive protein
EBER: EBV-encoded small RNA
EBV: Epstein–Barr virus
EBVaGC: gastric cancer patients with EBV infection
EBVnGC: gastric cancer patients without EBV infection
EDTA: ethylene di-amine tetra acetic acid
FFPE: formalin-fixed and paraffin-embedded
GC: gastric cancer
GGT: gamma-glutamyl transferase
GLOB: globulin
INR: international normalized ratio
IQR: interquartile range
LDH: lactate dehydrogenase
PCR: polymerase chain reaction
PLT: platelet
PT: prothrombin time
ROC: receiver operating characteristic
TBA: total bile acids
TBIL: total bilirubin
TT: thrombin time
WBC: white blood cell.
